# Genetic Ancestry and Population Structure Across Ecuador

**DOI:** 10.3390/genes17040437

**Published:** 2026-04-10

**Authors:** Fabricio González-Andrade

**Affiliations:** Postgraduate Studies Department in Health, Faculty of Health Sciences and Welfare, Indoamerica Technological University, Machala and Sabanilla Street, Quito 170301, Ecuador; fabriciogonzaleza@gmail.com

**Keywords:** Ecuador, genetic ancestry, population structure, three-way admixture, mitochondrial DNA, Y chromosome

## Abstract

**Background:** Ecuador is a genetically diverse population setting shaped by long-term interactions among Native American, European, and African populations across distinct ecological regions. Although multiple studies have examined ancestry patterns in Ecuadorian populations, the available evidence remains fragmented and methodologically heterogeneous. **Objective:** To systematically identify, critically appraise, and synthesize published studies on genetic ancestry and population structure in Ecuador. **Methods:** A systematic review was conducted in accordance with PRISMA 2020. Searches were performed in PubMed/MEDLINE, Scopus, Web of Science Core Collection, SciELO, and Google Scholar through 31 January 2026. Eligible studies reported extractable ancestry-related data from Ecuadorian populations using autosomal, mitochondrial DNA, Y-chromosomal, or other ancestry-relevant genetic markers. Methodological quality was assessed using an adapted Joanna Briggs Institute framework. Owing to substantial heterogeneity across marker systems, sampling strategies, and ancestry inference methods, findings were synthesized qualitatively rather than by meta-analysis. **Results:** Of 1243 records identified, 12 studies met the inclusion criteria. Across marker systems, the evidence consistently supported a three-way admixture framework involving Native American, European, and African ancestry components, together with substantial regional and population-specific heterogeneity. Autosomal studies generally showed higher Native American ancestry in Highland and Native American populations, whereas African ancestry was more prominent in Afro-Ecuadorian and some Coastal populations. Uniparental markers further supported persistent sex-biased admixture, with predominant Native American maternal lineages and comparatively greater European or African paternal contributions depending on region and population history. **Conclusions:** Ecuadorian populations share a broad three-way admixture framework, but with marked internal heterogeneity across regions and population groups. These findings highlight the importance of geographic and demographic context in ancestry interpretation and the need for larger, more balanced, and methodologically standardized genomic studies in Ecuador.

## 1. Introduction

Ecuador represents a genetically diverse population setting within Latin America, shaped by long-term demographic interactions among Native American, European, and African populations [1]. Broader studies of interethnic admixture in Latin America have shown that these processes have produced characteristic patterns of three-way admixture across the region [2]. Geographic analyses of Latin American Mestizo populations further demonstrate that ancestry proportions vary substantially across space and reflect local demographic history rather than uniform national profiles [3]. In Ecuador, one of the most consistently documented genomic signatures of these historical processes is sex-biased admixture, characterized by predominant Native American maternal lineages together with greater European or African paternal contributions depending on population history [4]. Similar ancestry gradients have also been described in broader Hispanic and Latin American populations, indicating that regional structure remains detectable even beyond national contexts [5].

Within this broader framework, Ecuador represents a particularly informative case because its population history reflects multiple forms of contact, migration, and admixture across distinct ecological regions. The country includes the Andean Highlands, the Pacific Coast, the Amazon Basin, and the Insular region, each associated with different demographic trajectories and historical patterns of interaction among Native American, European, and African populations [6]. Studies based on ancestry-informative markers in Ecuador’s principal population groups have supported a trihybrid origin while also showing marked variation among Mestizos, Native American, and Afro-Ecuadorians [7]. At the same time, population-genetic models indicate that ancestry inference is strongly influenced by marker density, reference population selection, and analytical methods, which limits direct comparability across studies [8]. This is particularly relevant in Ecuador, where studies using autosomal, mitochondrial, and Y-chromosomal data often capture different dimensions of demographic history [9].

Regional structure in Ecuador is also consistent with broader western South American patterns. Comparative evidence from Peru has shown that Andean and Amazonian populations may display substantial genomic differentiation despite geographic proximity [10]. These differences are important to interpret through transparent synthesis and reporting frameworks, and PRISMA 2020 provides an appropriate standard for systematic reviews in methodologically heterogeneous fields [11]. In parallel, conceptual work in human genetics has emphasized that ancestry should not be conflated with identity and that genetic essentialism should be avoided when interpreting socially complex populations [12]. This point is especially important in Ecuador, where research on genetic ancestry and ethnic identity has shown that self-identified ethnicity and genomic ancestry do not map onto one another in a simple or deterministic way [13]. Studies of genetic diversity in admixed Ecuadorian populations have further shown internal substructure within regional Mestizo groups, reinforcing the need for cautious interpretation of ancestry estimates [14].

Y-chromosomal analyses have contributed additional evidence of regional differentiation within Ecuador. A survey of Ecuador’s multi-ethnic population identified rare Native American founder lineages and provided further support for a tri-partite population structure [15]. Other Y-chromosomal work has shown a geographic mosaic of paternal ancestry across Highland, Coastal, and Amazonian populations [16]. Earlier studies in Afro-Ecuadorian communities from Esmeraldas documented strong African ancestry together with Native American maternal contributions, reinforcing the long-term demographic signature of sex-biased admixture in coastal Ecuador [17]. Beyond reconstructing demographic history, ancestry-related variation has also been examined in loci relevant to lactase persistence and pharmacogenomics, including LCT [18], DPYD [19], TPMT [20], and CYP2C19 [21]. These findings suggest that ancestry-aware approaches may also be relevant for biomedical and public health research in Ecuador.

Despite this growing body of work, the literature remains fragmented and methodologically heterogeneous. Recent narrative synthesis has emphasized Ecuador’s genetic mosaic and its implications for public health and precision medicine, but a systematic integration across marker systems and study designs has remained lacking [22]. This gap is especially relevant because evidence from neighboring Peru has shown that long-term demographic history can only be fully understood when genomic findings are interpreted within broader evolutionary and historical contexts [23]. More recent genomic initiatives in Peru have also highlighted the underrepresentation of many South American populations in global datasets and the need for more regionally grounded sampling strategies [24]. Similar concerns apply to Ecuador, where ancestry patterns are likely to remain incompletely understood unless underrepresented regions and populations are studied more systematically [13].

Accordingly, the aim of this systematic review was to identify, critically appraise, and synthesize published studies on genetic ancestry and population structure in Ecuador based on autosomal, mitochondrial, and Y-chromosomal evidence [11]. This review conceptualizes Ecuador’s genetic structure within a three-way admixture framework as a descriptive model rather than as evidence of population homogeneity [8]. The working hypothesis was that Ecuadorian populations would show a broadly consistent tri-continental ancestry pattern accompanied by substantial regional heterogeneity shaped by ecological stratification, historical asymmetries, and layered demographic processes [13]. By integrating findings across marker systems and study designs, this review seeks to provide a more coherent reference framework for interpreting Ecuador’s genomic diversity in anthropological, forensic, and biomedical contexts, including comparison with broader Hispanic and Latin American ancestry patterns [25].

## 2. Methods

### 2.1. Study Design and Reporting Framework

**Figure 1:**
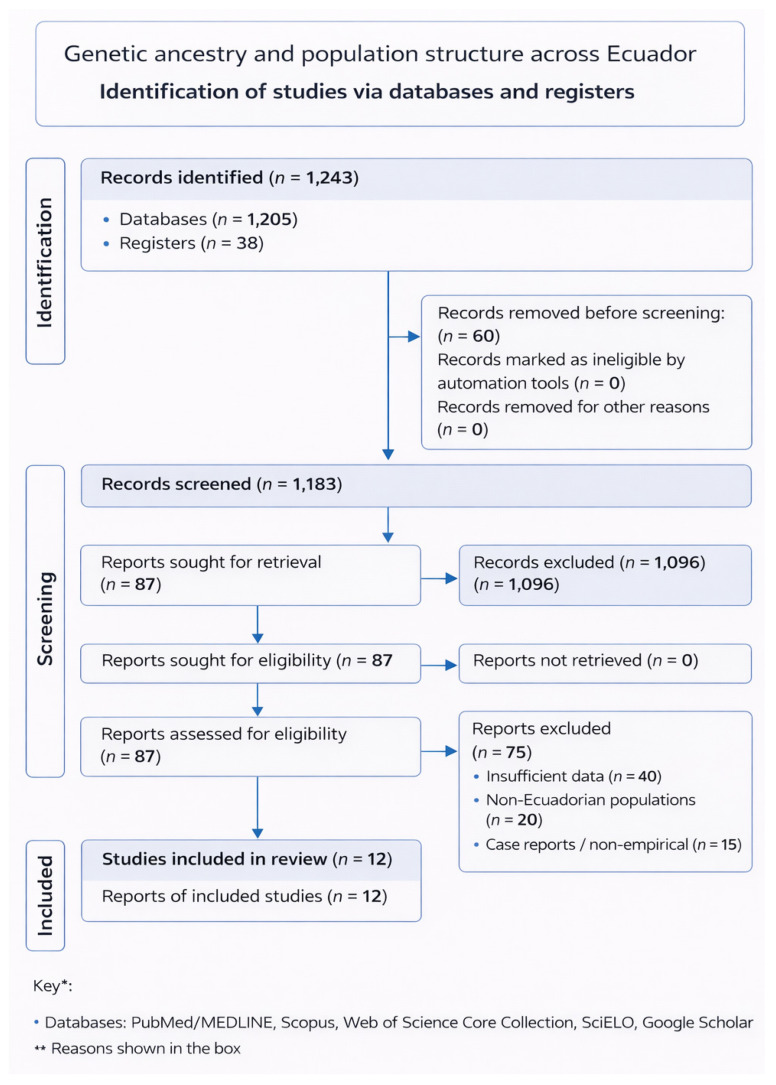
PRISMA 2020 flow diagram summarizing the study selection process for the systematic review of genetic ancestry and admixture in Ecuador, based on searches of bibliographic databases and registers only.

This study was conducted as a systematic review to identify, appraise, and synthesize published evidence on genetic ancestry and population structure in Ecuador. The review was conducted and reported in accordance with the PRISMA 2020 statement (Preferred Reporting Items for Systematic Reviews and Meta-Analyses). A completed PRISMA 2020 flow diagram summarizing the study selection process is presented in Figure 1. Because the available literature was expected to be methodologically heterogeneous in terms of marker systems, sampling strategies, and ancestry inference approaches, the review was designed from the outset as a structured qualitative synthesis rather than a quantitative meta-analysis. The objective was not to produce pooled national ancestry estimates, but to evaluate whether reproducible patterns of admixture and regional genetic structure could be identified across the included studies.

### 2.2. Protocol Development and Registration

A structured review protocol was developed a priori before literature screening and data extraction. The protocol specified the review question, eligibility criteria, information sources, search strategy, outcomes of interest, and planned approach to synthesis. This review was not prospectively registered in PROSPERO or another public repository, because it was designed as a descriptive synthesis of population genetic studies rather than an intervention review. No substantive deviations from the predefined protocol were introduced during study selection, extraction, appraisal, or synthesis.

### 2.3. Eligibility Criteria

Eligibility criteria were defined using a PICOS-informed framework adapted for population genetic research, recognizing that conventional clinical comparators are often not applicable in ancestry studies. Eligible studies included human populations sampled in Ecuador, including Mestizo, Native American, Afro-Ecuadorian, Montubio, and other regionally defined groups. To qualify for inclusion, studies had to report extractable ancestry-related data derived from at least one relevant marker system, including autosomal ancestry-informative markers, genome-wide autosomal SNPs, mitochondrial DNA haplogroups, Y-chromosomal STRs or SNPs, or ancestry-associated population comparisons judged relevant to the objectives of the review. Primary outcomes were autosomal ancestry proportions for Native American, European, and African components, as well as mtDNA and Y-chromosome haplogroup distributions. Secondary outcomes included measures of population structure such as principal component analysis, ADMIXTURE or STRUCTURE results, fixation indices, phylogenetic reconstructions, and other interpretable indicators of differentiation. Studies focused on Ecuadorian populations that provided ancestry-relevant genetic information were considered eligible. Reviews, editorials, commentaries, conference abstracts without extractable data, clinical studies without ancestry-relevant population data, and non-human genetic studies were excluded.

### 2.4. Information Sources

A comprehensive search strategy was implemented across multiple bibliographic databases to maximize coverage of published ancestry studies conducted in Ecuador. The databases searched were PubMed/MEDLINE, Scopus, Web of Science Core Collection, SciELO, and Google Scholar, and the final search was completed on 31 January 2026. These sources were selected to capture biomedical, anthropological, forensic, and regionally indexed Latin American literature. In addition to electronic database searches, the reference lists of all included studies and relevant reviews were manually screened to identify potentially eligible publications not captured through database retrieval. No restrictions were applied by year of publication. Studies published in English or Spanish were eligible.

### 2.5. Search Strategy

Search strategies were adapted to the syntax and indexing structure of each database using a combination of controlled vocabulary and free-text terms. Core search concepts included “Ecuador,” “Ecuadorian,” “genetic ancestry,” “admixture,” “population genetics,” “mitochondrial DNA,” “mtDNA,” “Y chromosome,” “haplogroup,” “ancestry-informative markers,” “AIM,” “AIM-SNP,” and “AIM-InDel.” An example PubMed strategy was: (Ecuador OR Ecuadorian) AND (genetic ancestry OR admixture OR population genetics OR mtDNA OR mitochondrial DNA OR Y-chromosome OR haplogroup OR AIM). Search terms were intentionally broad to capture studies using different marker systems and disciplinary labels. Because ancestry studies may be indexed inconsistently across biomedical, forensic, and anthropological sources, manual reference screening was used to improve retrieval.

### 2.6. Study Selection

All records identified from database searches and manual reference screening were imported into the review workflow and screened for eligibility. The search identified 1243 records across all sources. After removal of duplicates, titles and abstracts were screened against the predefined eligibility criteria. A total of 87 reports were retrieved for full-text assessment. Full texts were evaluated for relevance, extractable ancestry-related outcomes, adequacy of population description, and methodological eligibility. The most common reasons for exclusion were absence of ancestry-specific outcomes, lack of Ecuadorian population samples, insufficient methodological detail for extraction, and publication formats not suitable for data extraction, such as editorials, commentaries, or conference abstracts. An updated search through 31 January 2026, identified one additional publication; after full-text evaluation, this article was excluded because it did not provide extractable autosomal, mitochondrial, or Y-chromosomal ancestry data relevant to the review objectives. The final qualitative synthesis therefore included 12 studies: González-Andrade et al., 2007; Santangelo et al., 2017; Zambrano et al., 2019; Nagar et al., 2021; Flores-Espinoza et al., 2021; Villaescusa et al., 2021; Toscanini et al., 2018; Martínez-Labarga et al., 1999; Paz-Y-Miño et al., 2016; Farinango et al., 2022; Gallardo-Cóndor et al., 2023; and Alonso Llorente et al., 2024. The complete study selection process is summarized in the PRISMA 2020 flow diagram (Figure 1).

### 2.7. Data Extraction

Data extraction was conducted using a standardized and piloted template developed for this review. Extracted variables included publication details, year of publication, geographic region, population labels, sample size, sampling characteristics, marker systems, laboratory methods, ancestry inference approaches, and ancestry-related outcomes. Outcome data included autosomal ancestry proportions, mitochondrial DNA haplogroup distributions, Y-chromosome haplogroup frequencies, ancestry-associated allele frequency differences, and reported indicators of population structure such as principal component analysis, STRUCTURE, ADMIXTURE, fixation indices, and phylogenetic analyses. Data extraction was performed by a single reviewer. To reduce transcription and interpretation errors, extracted items were checked against the original full-text reports, and ambiguous variables were re-reviewed before inclusion in the synthesis. Although this verification process improved internal consistency, the absence of duplicate independent extraction remains a methodological limitation.

### 2.8. Risk of Bias and Methodological Quality Assessment

Methodological quality was assessed using an adaptation of the Joanna Briggs Institute Critical Appraisal Checklist for Analytical Cross-Sectional Studies, modified for population genetic research. Six appraisal domains were evaluated: (1) clarity of population definition, (2) adequacy and representativeness of sampling, (3) validity and discriminatory value of the genetic marker system, (4) appropriateness and transparency of reference populations used for ancestry inference, (5) reproducibility of laboratory and analytical procedures, and (6) completeness of outcome reporting. Each domain was rated as low concern, unclear concern, or great concern. Studies were classified overall as low risk of bias when no major concerns were identified, and most domains were adequately reported, moderate risk of bias when one or more domains were unclear or minor limitations were present, and high risk of bias when major concerns affected sampling, marker resolution, analytic transparency, or reporting completeness. Study-level judgments informed the interpretation of the findings.

### 2.9. Data Synthesis

Because the included studies differed substantially in marker systems, marker density, reference panels, ancestry estimation methods, and population sampling frameworks, a structured qualitative synthesis was performed instead of meta-analysis. The synthesis was organized into four domains: (1) autosomal ancestry patterns, (2) mitochondrial DNA lineages, (3) Y-chromosome lineages, and (4) regional or population-specific differentiation. All ancestry estimates reported in the review were extracted directly from the original publications; no recalculation, statistical harmonization, or weighted averaging was performed. Summary ranges were used descriptively to identify broad convergence across studies, but these values should not be interpreted as pooled national estimates. The purpose of the synthesis was therefore interpretive and comparative rather than quantitative.

### 2.10. Assessment of Heterogeneity and Rationale for Not Performing Meta-Analysis

Inter-study heterogeneity was assessed descriptively by examining differences in marker type, number of loci, reference population composition, ancestry inference method, population labels, and geographic sampling framework. Heterogeneity was also considered when evaluating whether ancestry estimates converged or diverged within broadly similar regional groups, while avoiding the assumption that all numerical estimates were directly comparable. A formal meta-analysis was not performed because the included studies varied substantially in design and did not satisfy the methodological assumptions required for valid statistical pooling. Ancestry estimates were generated using non-equivalent marker panels, distinct reference datasets, and different model-based analytical approaches. In addition, several studies focused on small or highly localized populations, including lineage-based analyses with limited external comparability. Under these conditions, pooled estimates would have risked overstating precision and obscuring meaningful methodological and biological differences. A qualitative synthesis was therefore considered the most rigorous and defensible approach. A formal assessment of reporting bias or publication bias was not performed because the included studies were few in number, methodologically heterogeneous, and not sufficiently comparable to support meaningful bias assessment across a common quantitative outcome.

### 2.11. Ethical Considerations and Patient or Public Involvement

This review was based exclusively on previously published studies and did not involve recruitment of new participants or generation of new genetic data. Accordingly, no additional institutional ethics approval was required for the present review. Ethical responsibility remained important, however, because the synthesis addressed ancestry, ethnicity, and population labels in a socially sensitive context. For that reason, ancestry estimates were interpreted as model-dependent statistical summaries rather than deterministic indicators of identity. No patients or members of the public were involved in the design, conduct, or reporting of this review. Future population genomic research in Ecuador would benefit from more structured community engagement, particularly with Native American and Afro-Ecuadorian populations, to support ethically grounded and culturally respectful research practices.

## 3. Results

### 3.1. Records Analyzed

The database search identified 1243 records across all information sources. After duplicate removal and title–abstract screening, 87 reports were retained for full-text assessment. Application of the predefined eligibility criteria resulted in 12 studies being included in the qualitative synthesis. The most common reasons for exclusion were the absence of ancestry-related outcomes, lack of Ecuadorian population samples, insufficient methodological reporting, or publication formats that did not allow data extraction, such as conference abstracts, editorials, or commentaries. An updated search conducted through 31 January 2026, identified one additional publication; however, it was excluded after full-text evaluation because it did not report extractable autosomal, mitochondrial, or Y-chromosomal ancestry data relevant to the objectives of this review. The final number of included studies therefore remained 12. The complete selection process is summarized in Figure 1.

### 3.2. Characteristics of Included Studies

The 12 included studies were published between 1999 and 2024 and showed substantial heterogeneity in scope, design, and analytical strategy. The included evidence comprised studies based on autosomal markers, genome-wide SNPs, mitochondrial DNA, Y-chromosomal markers, X-chromosomal markers, and ancestry-associated pharmacogenetic variants. Sample sizes ranged from relatively small lineage-focused or population-specific studies to several hundred participants in broader ancestry and population-structure analyses. Geographically, the Andean Highlands were the most frequently represented region, followed by the Pacific Coast and the Amazon Basin, whereas the Insular region remained minimally represented. Populations analyzed included Mestizos; Native American groups such as Highland and Amazonian Kichwas, Shuar, Waorani, and Tsáchilas; Afro-Ecuadorians, mainly from Esmeraldas and Imbabura; and Montubios from coastal agricultural regions.

**Figure 2:**
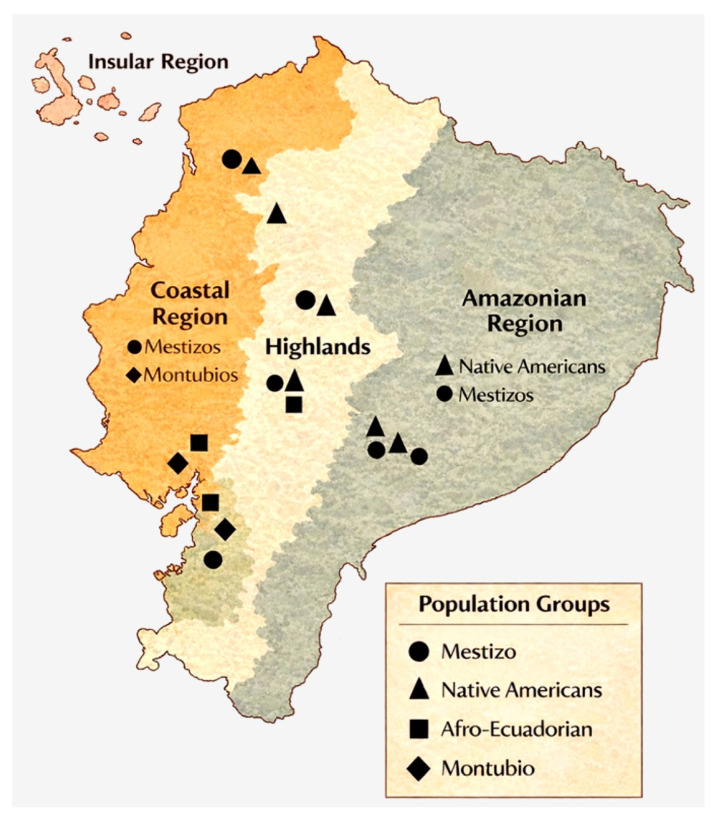
**Geographic distribution of population samples included in the systematic review across Ecuador’s major ecological regions.** Points indicate approximate sampling locations reported in the genetic studies included in the review. Symbols represent the main population groups analyzed, including Mestizo, Native American, Afro-Ecuadorian, and Montubio populations. The inset shows the Insular region (Galápagos Islands). Sampling locations correspond to study sites reported in the original publications and do not necessarily represent the birthplace, long-term residence, or broader geographic distribution of the participants.

The 12 studies included in the qualitative synthesis were: González-Andrade et al., 2007; Santangelo et al., 2017; Zambrano et al., 2019; Nagar et al., 2021; Flores-Espinoza et al., 2021; Villaescusa et al., 2021; Toscanini et al., 2018; Martínez-Labarga et al., 1999; Paz-Y-Miño et al., 2016; Farinango et al., 2022; Gallardo-Cóndor et al., 2023; and Alonso Llorente et al., 2024. Together, these studies contributed evidence on admixture patterns, maternal and paternal lineages, internal population structure, and ancestry-associated allele-frequency variation in Ecuadorian populations. The geographic distribution of sampled populations is shown in Figure 2.

### 3.3. Methodological Quality and Risk of Bias

Using the adapted Joanna Briggs Institute appraisal framework, two studies were classified as low risk of bias, nine as moderate risk, and one as high risk. Low-risk studies generally reported clearly defined populations, explicit sampling strategies, appropriate marker systems, and sufficiently transparent ancestry inference procedures, including specification of reference populations. Studies classified as moderate risk more often showed limitations such as restricted sample size, limited geographic representativeness, reduced marker resolution, partial reporting of recruitment procedures, or designs that were informative for ancestry interpretation but not directly comparable with broader population-structure studies. The single high-risk study was an older investigation with more limited methodological resolution and reduced comparability with more recent genomic studies. These differences were considered during synthesis, and findings from studies with greater methodological limitations were interpreted more cautiously. Despite this variability, recurrent descriptive ancestry patterns were observed across the included evidence base.

### 3.4. Autosomal Ancestry Patterns

Autosomal studies consistently supported a three-way admixture framework in Ecuadorian populations involving Native American, European, and African ancestry components. However, the relative contribution of each component varied substantially across regions and population groups. Highland Mestizo populations generally showed higher Native American ancestry, typically ranging from 55% to 70%, together with European ancestry of approximately 25% to 35% and lower African contributions. Coastal populations displayed more heterogeneous ancestry profiles, with Native American ancestry ranging from 40% to 55%, European ancestry from 30% to 45%, and African ancestry often reaching 10% to 20%. Afro-Ecuadorian populations showed predominantly African ancestry, frequently exceeding 60%, together with variable European and Native American contributions. Amazonian Native American populations showed the highest levels of Native American ancestry and the lowest levels of external admixture. Although ancestry estimates varied across studies, broadly similar regional patterns were observed, suggesting that the reported variability reflects both genuine population differentiation and methodological heterogeneity across datasets.

### 3.5. Mitochondrial DNA Haplogroups

Mitochondrial DNA analyses showed a predominance of Native American maternal lineages across most Ecuadorian populations. Haplogroups A2, B2, C1, and D1 accounted for most maternal lineages among Mestizo and Native American groups, often exceeding 80% to 95%, depending on region and sample size. Coastal populations showed higher frequencies of African mitochondrial haplogroups, particularly L0, L1, and L2, especially among Afro-Ecuadorians, who displayed predominantly African maternal ancestry. Native American mitochondrial haplogroups were also detected within Afro-Ecuadorian communities, which is consistent with previously described sex-biased admixture patterns. European maternal lineages were uncommon across populations and generally represented a small proportion of overall mitochondrial diversity. Taken together, these findings indicate strong Native American maternal continuity across multiple regions, together with localized African maternal contributions in coastal populations.

### 3.6. Y-Chromosome Haplogroups

Y-chromosome studies revealed patterns complementary to those observed for mitochondrial DNA and provided additional evidence of sex-specific demographic history. In Mestizo populations from both the Highlands and the Coast, European paternal haplogroups, particularly R1b and related western Eurasian lineages, were predominant and often accounted for 60% to 80% of Y-chromosomal diversity. Afro-Ecuadorian populations showed high frequencies of African Y-chromosome haplogroups, especially E1b1a, consistent with historical records of forced migration and settlement. Native American Y-chromosome haplogroups, primarily Q1a3a, were common among Amazonian and Highland Native American groups but occurred at lower frequencies in Mestizo populations. Additional studies also identified rare founder lineages and evidence of regional paternal differentiation, especially in Amazonian and multi-ethnic populations. Overall, Y-chromosomal evidence reinforced the persistence of asymmetry between maternal and paternal ancestry contributions across Ecuadorian populations.

### 3.7. Regional and Ethnic Differentiation

Clear regional and population-specific differentiation was observed across marker systems. Highland populations generally showed higher Native American ancestry and closer affinities with neighboring Andean groups. Coastal Mestizo populations showed lower Native American ancestry together with greater European and African contributions, consistent with region-specific demographic histories. Afro-Ecuadorian communities exhibited distinct African-derived genetic profiles and, in some historically isolated settings, relatively greater within-group homogeneity. Amazonian Native American populations displayed the most distinctive ancestry patterns, with minimal admixture in several groups and clear differentiation even among geographically proximate populations. Montubio populations showed admixture profiles broadly like those of Coastal Mestizos, consistent with shared regional histories. These findings underscore the importance of geography, local demographic history, and population-specific trajectories in shaping genetic diversity within Ecuador.

### 3.8. Overall Synthesis

Taken together, the results indicate that Ecuadorian populations share a broadly reproducible three-way admixture framework accompanied by substantial regional, population-specific, and lineage-specific heterogeneity. Autosomal and uniparental evidence converged in showing predominant Native American maternal ancestry, substantial European paternal contribution in many Mestizo populations, and regionally variable African ancestry, especially in Coastal and Afro-Ecuadorian groups. At the same time, the magnitude of ancestry components varied meaningfully across ecological regions and populations, indicating that national averages obscure important internal structure. The observed variability reflects both biological differentiation and study-level methodological differences in marker systems, sampling design, and analytical approaches. Overall, the available evidence supports a regionally stratified model of Ecuadorian genetic diversity shaped by long-term demographic processes rather than a uniform national ancestry profile. Table 1, Table 2 and Table 3 present, respectively, a summary of the key genetic ancestry studies conducted in Ecuador and included in the systematic review, the reported ranges of autosomal ancestry proportions across major Ecuadorian population groups, and the certainty of the evidence regarding ancestry patterns observed in Ecuadorian populations.

## 4. Discussion

### 4.1. Principal Findings and Contribution of the Review

This systematic review synthesizes the available evidence on genetic ancestry and population structure in Ecuador and shows that the country is best understood within a broadly consistent three-way admixture framework involving Native American, European, and African ancestral components. At the same time, the findings clearly indicate that this tri-continental pattern is not expressed uniformly across the country. Instead, the evidence supports a geographically structured and historically layered model of diversity, with substantial variation across the Highlands, Coast, Amazon, and, to a lesser extent, the Insular region. The main contribution of this review lies in integrating studies based on autosomal, mitochondrial, and Y-chromosomal markers under a single systematic framework. Rather than producing a pooled national estimate, this synthesis identifies recurrent demographic signals across heterogeneous studies. In doing so, it provides a more methodologically transparent overview of Ecuadorian population diversity than previous narrative accounts.

### 4.2. Internal Population Structure and the Limits of National Averages

One of the most robust conclusions of this review is that Ecuadorian genetic diversity cannot be adequately represented by a single national ancestry average. Across the included studies, Highland populations generally showed higher Native American ancestry, while Coastal populations exhibited more variable European and African contributions. Afro-Ecuadorian populations were characterized by stronger African ancestry signals, whereas Amazonian Native American groups showed the highest levels of Native American continuity and, in several studies, greater genetic differentiation. These findings indicate that ancestry in Ecuador is strongly shaped by geography, settlement history, and local demographic processes. As a result, national-level summaries may be useful for broad descriptive purposes but are insufficient for understanding fine-scale structure. This distinction is especially important in anthropological, biomedical, and forensic contexts, where internal population heterogeneity may be highly relevant. The data therefore support a mosaic model of Ecuadorian genetic diversity rather than a homogeneous national profile.

### 4.3. Comparison with Broader Latin American Patterns

When placed in the wider Latin American context, the ancestry patterns observed in Ecuador are broadly consistent with those reported in neighboring countries, particularly Peru. Studies from Peru have also documented marked differentiation along the Andes–Amazon divide, with stronger Native American continuity in highland and Amazonian populations and more substantial European and African contributions in coastal populations. Ecuador appears to follow a similar regional logic, suggesting that ecological boundaries, colonial demographic processes, and long-term population continuity have shaped genetic diversity across western South America in comparable ways. However, these similarities should be interpreted cautiously because direct cross-country numerical comparisons are limited by differences in marker systems, sample composition, and ancestry inference methods. At a broader continental scale, Ecuador also fits within the general Latin American pattern of tri-continental admixture, but with distinct internal substructure that reflects local historical trajectories. Thus, Ecuador is not an exception to regional demographic processes, but it does represent a particularly clear example of how those processes operate at fine geographic scale.

### 4.4. Methodological Heterogeneity and Interpretive Caution

A major strength of this review is that it does not attempt to conceal the substantial methodological heterogeneity of the available literature. The included studies differ in marker type, marker density, sampling design, reference population selection, and analytical strategy. Some studies rely on autosomal AIM panels, others on genome-wide SNP datasets, and others on uniparental markers or ancestry-associated pharmacogenetic loci. Under these conditions, ancestry estimates should not be interpreted as directly equivalent numerical measurements across studies. Instead, they should be understood as model-dependent statistical estimates generated under different methodological assumptions. For this reason, the present review did not perform a formal meta-analysis, and the ancestry ranges summarized in the tables should be interpreted as descriptive patterns rather than pooled estimates. The convergence observed across studies is therefore meaningful at the level of broad demographic trends, not at the level of strict quantitative comparability. This distinction is essential to avoid overinterpretation and to maintain methodological rigor.

The interpretation of the findings should be considered considering the methodological heterogeneity documented across the included studies. The Supplementary Materials were intended to improve transparency in this regard: Supplementary Table S1 details the database-specific search strategies, Supplementary Table S2 provides study-level risk-of-bias assessments, and Supplementary Table S3 summarizes the principal methodological features affecting comparability across studies. Taken together, these materials support the decision to conduct a structured qualitative synthesis rather than formal meta-analysis and help clarify both the strengths and the limitations of the current evidence on genetic ancestry and population structure in Ecuador.

### 4.5. Sex-Biased Admixture and Historical Interpretation

Another consistent finding across the reviewed literature is the asymmetry between maternal and paternal ancestry contributions. Mitochondrial DNA studies repeatedly show strong Native American maternal continuity across many Ecuadorian populations, while Y-chromosome studies more often reveal greater European or African paternal contributions depending on region and population history. This pattern is consistent with long-recognized sex-biased admixture in Latin America and reflects the demographic consequences of colonial expansion, enslavement, forced migration, labor systems, and unequal social relations. In Ecuador, these processes appear to have left stable genomic signatures that remain detectable across different marker systems. Interpreting these findings historically is important because it prevents a purely biological reading of admixture patterns. The observed asymmetries do not describe fixed identities, but rather the long-term imprint of social and demographic processes. In that sense, genomic evidence contributes to historical reconstruction while also requiring careful contextual interpretation.

### 4.6. Ancestry, Identity, and the Avoidance of Genetic Essentialism

This review also underscores the need to distinguish clearly between genetic ancestry and social identity. Categories such as Mestizo, Native American, Afro-Ecuadorian, and Montubio are historically and socially shaped classifications and do not correspond in a simple or deterministic way to genomic ancestry proportions. Several studies conducted in Ecuador suggest that self-identified ethnicity and measured ancestry may diverge, particularly in socially complex and regionally mixed populations. For that reason, ancestry estimates should be interpreted as statistical descriptions of genetic similarity relative to selected reference populations, not as biological definitions of identity or belonging. Maintaining this distinction is especially important in human population genetics, where genomic findings can easily be overextended into social or political claims. The present synthesis adopts a deliberately cautious framework to avoid genetic essentialism and to support interpretation that is historically informed, socially responsible, and conceptually precise. This is particularly relevant in Ecuador, where cultural identity, language, territory, and historical experience remain central dimensions of population diversity.

### 4.7. Potential Contribution and Limitations of Direct-to-Consumer Ancestry Databases

Although this review focused exclusively on peer-reviewed academic studies, it is reasonable to consider whether direct-to-consumer ancestry databases such as 23andMe or Ancestry might contribute useful information on Ecuadorian population structure. In principle, such databases may provide access to large numbers of individuals with partial Ecuadorian ancestry, including members of diaspora populations, and may eventually help identify broad patterns of shared ancestry segments. However, these platforms were not included in the present review because they do not constitute standardized peer-reviewed population studies, their sampling is self-selected rather than population-based, and their ancestry algorithms and reference panels are often proprietary or only partially transparent. For these reasons, their outputs are not directly comparable with the academic literature synthesized here. At present, such databases may be useful as supplementary exploratory resources, but they cannot substitute for systematically designed genomic studies with transparent methodology, defined sampling strategies, and reproducible analytical procedures.

### 4.8. Implications for Biomedical Research and Public Health

The findings of this review have implications beyond population history. Regional and population-specific ancestry differences are relevant for biomedical research, pharmacogenomics, and precision medicine because allele frequencies at clinically important loci may vary across ancestry backgrounds. In Ecuador, studies of loci such as LCT, DPYD, TPMT, and CYP2C19 suggest that ancestry-related variation may have practical implications for drug metabolism and other health-related outcomes. However, it is important not to overstate this connection. Genetic ancestry should not be used as a substitute for direct genotyping, nor should it be treated as a deterministic predictor of individual disease risk or treatment response. Instead, the main implication is that biomedical research in Ecuador should avoid assuming genetic homogeneity and should incorporate ancestry-aware sampling and analysis strategies. This is especially important in a country where internal population structure is substantial and where underrepresented groups have historically been excluded from genomic research.

### 4.9. Limitations of the Review

Several limitations should be considered when interpreting the findings of this systematic review. First, although a structured protocol was developed before the review began, it was not prospectively registered in PROSPERO or another public repository, which limits external verification of pre-specified methods. Second, study selection, data extraction, and risk-of-bias assessment were conducted by a single reviewer. Although standardized templates and repeated verification against the original studies were used to reduce error, the absence of duplicate independent review may have introduced subjective judgments or undetected inaccuracies. Third, the included studies were highly heterogeneous in sample size, marker systems, ancestry inference methods, and geographic coverage, which limited direct comparability and precluded meta-analysis. Fourth, some populations, particularly several Amazonian Native American groups and the Galápagos region, remain underrepresented in the available literature. Finally, only a subset of studies could be included in descriptive autosomal comparison tables because many studies used non-comparable marker systems or did not report directly interpretable autosomal ancestry proportions. These limitations do not invalidate the review, but they do define the scope of the inferences that can reasonably be drawn. Finally, certainty of evidence was evaluated descriptively rather than through a formal framework such as GRADE, which should be considered when interpreting the qualitative certainty ratings presented in this review.

### 4.10. Evidence Gaps and Future Directions

Despite the number of studies identified, important gaps remain in the genomic characterization of Ecuadorian populations. Future research should prioritize larger and more geographically balanced genome-wide datasets that include the Highlands, Coast, Amazon, and Insular region in a more systematic way. Particular attention should be given to underrepresented Native American populations in the Amazon, Afro-descendant communities outside the most frequently studied localities, and populations for which only small or single-study datasets currently exist. Studies that integrate autosomal, mitochondrial, and Y-chromosomal markers in the same individuals would provide a more complete picture of demographic history and sex-biased admixture. Greater standardization is also needed in reference panel selection, ancestry inference methods, uncertainty reporting, and description of recruitment procedures. In addition, future research should incorporate interdisciplinary collaboration with anthropology, linguistics, archeology, and history to contextualize genomic findings more responsibly. Meaningful community engagement and equitable benefit sharing should also become central components of population genomics research in Ecuador.

### 4.11. Concluding Perspective

In summary, the evidence reviewed here indicates that Ecuadorian populations share a broad three-way admixture framework, but that this framework is expressed through pronounced regional and population-specific heterogeneity. The most reproducible signals across studies are strong Native American maternal continuity, variable but often substantial European paternal contribution in Mestizo populations, regionally concentrated African ancestry in Coastal and Afro-Ecuadorian populations and marked internal differentiation across ecological regions. These findings reinforce the importance of interpreting ancestry estimates within their methodological, geographic, and historical context. Ecuador should therefore be understood not as a single genomic entity, but as a complex and internally structured population landscape shaped by long-term demographic processes. By emphasizing transparency, caution, and regional specificity, this review provides a more solid foundation for future research in population genomics, ancestry-informed medicine, and the ethical interpretation of human genetic diversity in Ecuador.

## 5. Conclusions

This systematic review synthesizes two decades of published research on genetic ancestry and population structure in Ecuador and shows that the available evidence is broadly consistent with a three-way admixture framework involving Native American, European, and African ancestral components. At the same time, the findings demonstrate that this framework is expressed through substantial regional and population-specific heterogeneity across the Highlands, Coast, Amazon, and, to a lesser extent, the Insular region. Ecuadorian genetic diversity is therefore better understood as a geographically structured mosaic than as a single national ancestry profile. The convergence observed across autosomal, mitochondrial, and Y-chromosomal studies supports the interpretation that long-term demographic history, sex-biased admixture, and ecological stratification have played central roles in shaping the country’s present-day population structure.

These conclusions should be interpreted considering the methodological limitations of the available literature and of the review itself. The included studies differed substantially in marker systems, sample composition, reference populations, and ancestry inference methods, which precluded formal meta-analysis and limited direct cross-study comparability. In addition, several populations remain underrepresented, particularly some Amazonian Native American groups and the Insular region, and the review protocol was not prospectively registered. Study selection, data extraction, and risk-of-bias assessment were also conducted by a single reviewer, which may have increased the possibility of undetected errors or subjective judgment. Accordingly, the ancestry patterns summarized here should be understood as descriptive syntheses of the published evidence rather than as precise pooled estimates of national ancestry proportions.

Despite these limitations, this review provides the first systematic and methodologically explicit synthesis of Ecuadorian ancestry studies across multiple marker systems. The findings highlight the importance of avoiding assumptions of genetic homogeneity in anthropological, forensic, and biomedical contexts and underscore the need for larger, better-balanced, and more methodologically standardized genomic studies in Ecuador. Future research should prioritize genome-wide sampling across underrepresented regions and populations, improve transparency in ancestry inference methods, and strengthen interdisciplinary and community-engaged approaches to interpretation. By integrating fragmented evidence into a coherent and cautious framework, this review offers a useful reference point for future population genomics and ancestry-informed biomedical research in Ecuador.

## Figures and Tables

**Table 1:** **Summary of key genetic ancestry studies conducted in Ecuador included in the systematic review.** This table summarizes the principal characteristics of the studies included in the review, including sampled populations, approximate sample sizes, marker systems, and the main findings related to ancestry patterns and population structure in Ecuador. Studies based on autosomal, mitochondrial DNA, and Y-chromosomal markers are included, together with selected ancestry-associated pharmacogenetic studies that contributed relevant population-level information. Because the included studies differed substantially in marker type, sampling strategy, and analytical design, the findings should be interpreted as descriptive summaries rather than directly comparable quantitative estimates.

Author/Year (Ref)	Population(s)	Sample Size	Genetic Markers Used	Main Findings
González-Andrade et al., 2007 [4]	Mestizos, Native Americans (Kichwas), Afro-Ecuadorians	*n* ≈ 300	Autosomal STRs, mtDNA, Y-STRs	Strong sex-biased admixture, with predominance of European Y-chromosome lineages and Native American mtDNA in Mestizos; Afro-Ecuadorians showed African paternal ancestry together with substantial Native American autosomal contribution.
Santangelo et al., 2017 [7]	Kichwas, Mestizos, Afro-Ecuadorians	*n* ≈ 150	AIM-SNPs (Precision ID Panel)	Confirmed Ecuador’s tri-hybrid genetic structure; Kichwas clustered more closely with Native American ancestry, whereas Mestizos and Afro-Ecuadorians showed more diverse admixture profiles.
Zambrano et al., 2019 [9]	Mestizos (multi-regional)	*n* ≈ 200	AIMs-InDels, autosomal SNPs, mtDNA, Y chromosome	Reported average ancestry proportions of approximately 51% Native American, 38% European, and 7% African, with broad concordance across autosomal and uniparental markers.
Nagar et al., 2021 [13]	Mestizos, Afro-Ecuadorians, Montubios, Tsáchilas	*n* ≈ 400	Genome-wide SNPs	Mestizos showed approximately 45–70% Native American, 20–40% European, and 5–15% African ancestry; Afro-Ecuadorians and Tsáchilas displayed distinct ancestry profiles and evidence of internal substructure.
Flores-Espinoza et al., 2021 [14]	Mestizos (multiple regions)	*n* = 586	X-InDels, X-STRs	Identified substructure within Mestizo populations and strong regional genetic stratification, consistent with internal migration and regional demographic history.
Villaescusa et al., 2021 [15]	Amazonian Kichwas, Highland Kichwas, Coastal populations	*n* = 527	Y-SNPs, Y-STRs	Detected the rare Native American Y-lineage C3-MPB373, reaching up to 28% in Amazonian Kichwas, and provided evidence of founder effects and deep paternal divergence across regions.
Toscanini et al., 2018 [16]	Coastal, Highland, Amazonian populations	*n* = 415	Y-STRs (Yfiler, PPY23)	Revealed a geographic mosaic of paternal ancestry, with predominance of European Y lineages in the Highlands, African lineages on the Coast, and Native American lineages in the Amazon.
Martínez-Labarga et al., 1999 [17]	Afro-Ecuadorians (Esmeraldas)	*n* ≈ 120	Autosomal STRs, mtDNA	Afro-Ecuadorians showed high African autosomal ancestry but predominantly Native American maternal lineages, supporting sex-biased admixture.
Paz-Y-Miño et al., 2016 [18]	Mestizos, Native Americans, Afro-Ecuadorians	*n* ≈ 200	LCT variant (rs4988235)	The European-derived LCT-13910*T allele was most frequent in Mestizos and least frequent in Native American groups, consistent with variation associated with European ancestry proportion.
Farinango et al., 2022 [19]	Mestizos, Afro-Ecuadorians, Native Americans, Montubios	*n* ≈ 300	DPYD variants + AIMs	DPYD pharmacogenomic variants were strongly associated with individual ancestry proportions, supporting the relevance of ancestry-informed precision medicine in Ecuador.
Gallardo-Cóndor et al., 2023 [20]	Afro-Ecuadorians, Mestizos, Native Americans, Montubios	*n* = 550	TPMT variants + AIMs	TPMT deficiency alleles showed population-specific distributions associated with ancestry background, supporting population-tailored pharmacogenetic interpretation.
Alonso Llorente et al., 2024 [21]	Mestizos, Afro-Ecuadorians, Native Americans	*n* ≈ 250	CYP2C19 variants	CYP2C19 pharmacogenetic variants showed clear ancestry-related frequency differences with potential implications for personalized therapy.

**Note:** LCT, DPYD, TPMT, and CYP2C19 are autosomal loci. These studies were included because they reported ancestry-relevant population differences in Ecuadorian groups, although they were not designed as primary mitochondrial DNA or Y-chromosomal ancestry studies.

**Table 2:** Reported autosomal ancestry proportion ranges across major Ecuadorian population groups. Values represent descriptive ranges extracted from autosomal studies included in the qualitative synthesis. Proportions correspond to Native American, European, and African ancestry components as reported in the original publications. No statistical pooling or meta-analysis was performed, and the values should not be interpreted as harmonized national estimates.

Population/Region	Native American (%)	European (%)	African (%)	Total N (Range)	Source Studies
Highland Mestizo	55–70	25–35	3–10	80–450	[7,9,13,14]
Coastal Mestizo	40–55	30–45	10–20	70–380	[7,9,14]
Afro-Ecuadorian (Esmeraldas, Imbabura)	10–30	15–30	60–80	60–250	[4,7,9,17]
Amazonian Native American	80–98	0–10	0–5	40–220	[9,13]
Highland Native American (Kichwa, Tsáchila)	75–95	0–15	0–5	50–200	[4,9,13]
Montubio (Coastal agricultural regions)	45–60	30–40	5–15	65–180	[7,9]

**Note:** Ranges are presented for descriptive comparison only. Direct numerical comparability across studies is limited by differences in marker density, reference populations, sampling strategies, and ancestry inference methods.

**Table 3:** **Certainty of evidence for ancestry patterns observed in Ecuadorian populations.** This table summarizes the certainty of evidence for major ancestry patterns identified across Ecuadorian population groups. Certainty judgments reflect descriptive convergence across independent datasets, replication across marker systems, sample size considerations, geographic representation, and methodological transparency of the included studies. Because the evidence base is heterogeneous and the review did not perform statistical pooling, these ratings should be interpreted as qualitative judgments intended to support interpretation rather than as formal quantitative evidence grades.

Population/Group	Reported Ancestry Pattern (Descriptive Range)	Certainty of Evidence	Evidence Base (References)	Key Interpretation
Highland Mestizos	NAM: 55–70%; EUR: 25–35%; AFR: 3–10%	Moderate	[7,9,13,14]	The available evidence supports a consistent three-way admixture pattern with higher Native American ancestry than in Coastal Mestizo populations. This interpretation is supported by autosomal AIM panels, genome-wide SNP datasets, and X-chromosomal analyses.
Coastal Mestizos	NAM: 40–55%; EUR: 30–45%; AFR: 10–20%	Low–moderate	[7,9,14]	The available studies suggest a greater African contribution than in Highland Mestizo populations. However, regional heterogeneity and methodological differences limit confidence in direct quantitative comparison.
Highland Native American populations (Kichwas, Tsáchilas)	NAM: 75–95%; EUR: 0–15%; AFR: 0–5%	Moderate	[4,9,13]	The evidence supports strong Native American continuity with limited European and African admixture. This interpretation is strengthened by convergence across autosomal and uniparental evidence, although direct comparability remains limited across marker systems.
Amazonian Native American populations (Kichwas, Waorani, Shuar)	NAM: 80–98%; EUR: 0–10%; AFR: 0–5%	Low–moderate	[9,13]	The available data indicate strong Native American ancestry and relative genetic isolation in several Amazonian populations. Certainty remains limited because the number of studies is small and genome-wide evidence is still restricted.
Tsáchilas (distinct analyses)	NAM: 60–75%; EUR: 20–30%; AFR: 5–15%	Low	[13]	The available evidence suggests a tri-continental admixture profile with internal substructure. However, certainty remains low because the finding is based on limited independent replication.
Afro-Ecuadorians (Esmeraldas and Chota Valley)	AFR: 60–80%; NAM: 10–30%; EUR: 10–25%	Moderate	[4,7,9,17]	The evidence consistently supports predominantly African ancestry together with persistent Native American maternal contribution. This pattern has been documented across autosomal STRs, AIM-based studies, and mitochondrial DNA analyses.
Montubios (Coastal agricultural regions)	NAM: 45–60%; EUR: 30–45%; AFR: 5–15%	Low–moderate	[7,9,13]	The available evidence suggests an admixture profile broadly like that of Coastal Mestizo populations. Certainty is reduced by the limited number of independent studies and variation in analytical design.

**Note:** Certainty categories were assigned descriptively based on study quality, replication, sample size, geographic coverage, and consistency of ancestry patterns across datasets. These categories do not represent formal GRADE-based assessments.

## Data Availability

All data synthesized in this systematic review were obtained from previously published studies. No new primary data were generated. Extracted datasets, PRISMA flow information, and Supplementary Materials are available from the corresponding author upon reasonable request.

## Supplementary Materials

The following supporting information can be downloaded at https://www.mdpi.com/article/10.3390/genes17040437/s1, Table S1: Full database-specific search strategies used in the systematic review; Table S2: Risk-of-bias assessment of studies included in the qualitative synthesis; Table S3: Methodological characteristics affecting comparability across included studies.

## Abbreviations

The following abbreviations are used in this manuscript:

AIMs	Ancestry-informative markers
AIM-InDels	Ancestry-informative insertions/deletions
AIM-SNPs	Ancestry-informative single-nucleotide polymorphisms
mtDNA	Mitochondrial DNA
Y-STRs	Y-chromosome short tandem repeats
Y-SNPs	Y-chromosome single-nucleotide polymorphisms
SNPs	Single-nucleotide polymorphisms
STRs	Short tandem repeats
PRISMA	Preferred Reporting Items for Systematic Reviews and Meta-Analyses
