# Supplementary material for: Genetic Ancestry and Population Structure Across Ecuador
Source: Genes (Basel). 2026 Apr 10;17(4):437. doi: 10.3390/genes17040437 (PMC13116061; doi:10.3390/genes17040437)
Supplement: Supplementary file 1 [file genes-17-00437-s001.zip › genes-4258426-supplementary.pdf]

Table S1. Full database-specific search strategies used in the systematic review. Searches were performed through January 31, 2026. Search syntax was adapted to the indexing structure and operators supported by each database.

Database	Search strategy	Filters / limits applied
PubMed/MEDLINE	(Ecuador OR Ecuadorian) AND (genetic ancestry OR admixture OR population genetics OR mtDNA OR mitochondrial DNA OR Y-chromosome OR haplogroup OR AIM*)	No date restriction; English or Spanish
Scopus	TITLE-ABS-KEY (Ecuador OR Ecuadorian) AND TITLE-ABS-KEY ("genetic ancestry" OR admixture OR "population genetics" OR mtDNA OR "mitochondrial DNA" OR "Y chromosome" OR haplogroup OR "ancestry-informative markers" OR AIM OR "AIM-SNP" OR "AIM-InDel")	No date restriction; English or Spanish
Web of Science Core Collection	TS= (Ecuador OR Ecuadorian) AND TS= ("genetic ancestry" OR admixture OR "population genetics" OR mtDNA OR "mitochondrial DNA" OR "Y chromosome" OR haplogroup OR "ancestry-informative markers" OR AIM OR "AIM-SNP" OR "AIM-InDel")	No date restriction; English or Spanish
SciELO	(Ecuador OR Ecuadorian) AND ("genetic ancestry" OR admixture OR "population genetics" OR mtDNA OR "mitochondrial DNA" OR "Y chromosome" OR haplogroup OR "ancestry-informative markers" OR AIM OR "AIM-SNP" OR "AIM-InDel")	No date restriction; English or Spanish
Google Scholar	("Ecuador" OR "Ecuadorian") AND ("genetic ancestry" OR admixture OR "population genetics" OR mtDNA OR "mitochondrial DNA" OR "Y chromosome" OR haplogroup OR AIM)	No date restriction; English or Spanish; screened for relevance
Manual reference screening	Reference lists of included studies and relevant reviews were screened manually to identify additional eligible publications not captured electronically	Not applicable

Note: Core search concepts included "Ecuador," "Ecuadorian," "genetic ancestry," "admixture," "population genetics," "mitochondrial DNA," "mtDNA," "Y chromosome," "haplogroup," "ancestry-informative markers," "AIM," "AIM-SNP," and "AIM-InDel." Database syntax was adapted as required by each platform.

Table S2. Risk-of-bias assessment of studies included in the qualitative synthesis. Risk-of-bias judgments were assigned using the adapted appraisal framework described in the Methods section. Studies were classified as low, moderate, or high risk of bias according to sampling quality, marker suitability, analytical transparency, and completeness of reporting.

Study	Main methodological limitations	Overall risk of bias	Reason for overall judgment
González-Andrade et al., 2007 [4]	Older study design; limited comparability with more recent genomic datasets	Moderate	Relevant multi-marker study with clear ancestry-related findings, but with lower methodological resolution than more recent studies.
Santangelo et al., 2017 [7]	Limited sample size	Low–moderate	Clear AIM-based ancestry analysis with interpretable results and adequate reporting, although the limited sample size reduces confidence relative to larger or higher-resolution studies.
Zambrano et al., 2019 [9]	Minor limitations in cross-study comparability	Low	Strong autosomal and uniparental design with directly interpretable ancestry estimates and adequate analytical transparency.
Nagar et al., 2021 [13]	Minor limitations in geographic representativeness	Low	Genome-wide study with strong analytical value, clear contribution to ancestry inference, and adequate methodological reporting.
Flores-Espinoza et al., 2021 [14]	X-chromosomal focus limits direct comparability with some autosomal studies	Low–moderate	Well-reported study with valuable regional insights, although the X-chromosomal design limits direct comparability with autosomal ancestry studies.
Villaescusa et al., 2021 [15]	Y-chromosomal design limits comparability with autosomal ancestry estimates	Moderate	Informative paternal-lineage study with good reporting but limited for overall ancestry comparison.
Toscanini et al., 2018 [16]	Y-chromosomal design limits comparability with autosomal ancestry estimates	Moderate	Useful regional paternal-lineage study, but not directly comparable with autosomal studies.
Martínez-Labarga et al., 1999 [17]	Older study; smaller sample; limited methodological resolution	High	Historically relevant study, but with lower analytical detail and lower comparability than more recent studies.
Paz-Y-Miño et al., 2016 [18]	Single-locus design	Moderate	Relevant ancestry-associated study, but not a full ancestry inference design.
Farinango et al., 2022 [19]	Pharmacogenetic focus; limited comparability with population ancestry studies	Moderate	Useful ancestry-associated evidence, but not designed as a primary ancestry structure study.
Gallardo-Cóndor et	Pharmacogenetic focus; limited comparability	Moderate	Good reporting, but not directly comparable with core ancestry studies.

al., 2023 [20]	with population ancestry studies		
Alonso Llorente et al., 2024 [21]	Pharmacogenetic focus; limited comparability with population ancestry studies	Moderate	Interethnic pharmacogenetic study with ancestry relevance, but not a primary population structure study.

Table S3. Methodological characteristics affecting comparability across included studies. This table summarizes key methodological features that influenced cross-study comparability in the review, including marker systems, analytical approaches, and reference population reporting. It supports the rationale for conducting a structured qualitative synthesis instead of formal meta-analysis.

Study	Marker system	Approximate marker resolution	Reference populations reported	Main ancestry inference approach	Main comparability limitation
González-Andrade et al., 2007 [4]	Autosomal STRs, mtDNA, Y-STRs	Low to moderate	Limited / study-specific	Multi-marker ancestry interpretation	Older marker framework and limited comparability with genome-wide datasets.
Santangelo et al., 2017 [7]	AIM-SNPs	Moderate	Yes	AIM-based ancestry estimation	Reduced marker density relative to genome-wide studies.
Zambrano et al., 2019 [9]	AIMs-InDels, autosomal SNPs, mtDNA, Y chromosome	Moderate	Yes	Comparative autosomal and uniparental ancestry estimation	Mixed marker framework limits direct comparison with single-platform studies.
Nagar et al., 2021 [13]	Genome-wide SNPs	High	Yes	Genome-wide ancestry and population structure analysis	Different analytical scale relative to reduced-marker studies.
Flores-Espinoza et al., 2021 [14]	X-InDels, X-STRs	Moderate	Limited / study-specific	X-chromosomal population structure analysis	X-chromosomal design is not directly equivalent to autosomal ancestry estimation.
Villaescusa et al., 2021 [15]	Y-SNPs, Y-STRs	Moderate	Not applicable / lineage-based	Paternal lineage and haplogroup analysis	Uniparental design does not provide directly comparable autosomal ancestry proportions.

Toscanini et al., 2018 [16]	Y-STRs	Moderate	Not applicable / lineage-based	Paternal ancestry structure analysis	Y-chromosomal design does not provide directly comparable autosomal ancestry proportions.
Martínez-Labarga et al., 1999 [17]	Autosomal STRs, mtDNA	Low	Limited / older study design	Population structure and maternal ancestry interpretation	Older methodological framework and lower resolution than more recent studies.
Paz-Y-Miño et al., 2016 [18]	LCT locus	Single locus	Not applicable	Allele frequency comparison	Single-locus design is not suitable for direct ancestry proportion comparison.
Farinango et al., 2022 [19]	DPYD variants + AIMs	Low to moderate	Yes	Pharmacogenetic association with ancestry proportions	Primary focus was pharmacogenetics rather than population ancestry structure.
Gallardo-Cóndor et al., 2023 [20]	TPMT variants + AIMs	Low to moderate	Yes	Pharmacogenetic association with ancestry proportions	Primary focus was pharmacogenetics rather than population ancestry structure.
Alonso Llorente et al., 2024 [21]	CYP2C19 variants	Single / few loci	Limited / not primary ancestry design	Interethnic allele frequency comparison	Not designed as a full ancestry inference study.

Note: Marker resolution is described qualitatively to highlight broad differences in methodological scale across studies. This table is intended to support interpretation of inter-study heterogeneity and the rationale for qualitative synthesis.
